# Determination of the Volatile Profile of Lemon Peel Oils as Affected by Rootstock

**DOI:** 10.3390/foods9020241

**Published:** 2020-02-24

**Authors:** Marlene G. Aguilar-Hernández, Paola Sánchez-Bravo, Francisca Hernández, Ángel A. Carbonell-Barrachina, Joaquín J. Pastor-Pérez, Pilar Legua

**Affiliations:** 1 Departamento de Horticultura, Universidad Nacional Agraria La Molina, Av. La Molina s/n, Lima 15026, Peru; maguilarhe@lamolina.edu.pe; 2 Departamento Tecnología Agroalimentaria, Grupo Calidad y Seguridad Alimentaria, Escuela Politécnica Superior de Orihuela, Universidad Miguel Hernández de Elche, Carretera de Beniel, Km 3.2, 03312 Orihuela, Spain; paola.sb94@gmail.com (P.S.-B.); angel.carbonell@umh.es (Á.A.C.-B.); 3 Departamento de Producción Vegetal y Microbiología, Grupo Producción Vegetal, Escuela Politécnica Superior de Orihuela, Universidad Miguel Hernández de Elche, Carretera de Beniel, km 3.2, 03312 Orihuela, Spain; p.legua@umh.es; 4 Departamento de Ingeniería Agroforestal, Escuela Politécnica Superior de Orihuela, Universidad Miguel Hernández de Elche, Carretera de Beniel, km 3.2, 03312 Orihuela, Spain; jjpastor@umh.es

**Keywords:** aroma composition, *Citrus limon* (L.), concentrations—monoterpene, GC-MS, limonene, sesquiterpenes, aldehydes

## Abstract

*Citrus limon* (L.) Burm is an important crop that grows between latitudes 30° North and 30° South, the main producers being China, the USA, Mexico, India, Brazil, and Spain. In Spain, lemon grows mainly in Mediterranean areas such as Murcia, Valencia, and Andalucía. The most cultivated varieties are “Fino” and “Verna”. In this study, five varieties of lemon, “Verna”, “Bétera”, “Eureka”, “Fino 49”, and “Fino 95” were evaluated on different rootstocks: three new Forner-Alcaide (“FA13”, “FA5”, “FA517”), *Citrus macrophylla,* Wester, and *Citrus aurantium* L. Hydrodistillation was used to obtain essential oil from fresh peels and then the volatile profile was studied by gas chromatography-mass spectrometry (GC-MS). A total of 26 volatile compounds were identified, limonene being the main one followed by β-pinene, γ-terpinene, sabinene, and α-pinene. The results revealed that Forner-Alcaide rootstocks (“FA5” > “FA517” > “FA13”) proved to be the best rootstocks for the aroma quality as they led to high volatile contents, followed by *C. aurantium* and *C. macrophylla*. Among the other varieties, the most aromatic one was “Eureka”. The whole trend was as follows (in decreasing order): “Eureka” > “Bétera” > “Fino 95” > “Verna” > “Fino 49”.

## 1. Introduction

Lemon is an important crop that grows in different parts of the world. The main lemon producers are in China, the United States, Mexico, India, Brazil, and Spain [1]. In Spain, it grows mainly in the Mediterranean areas of Murcia, Valencia, and Andalucía, which represent the highest productions [2]. These high productions are associated with selected, suitable, and compatible rootstocks [3]. Moreover, the use of rootstock influences quantitative and qualitative characteristics of agronomic variables which improve size, color, soluble solids, acidity, yield, and quality of the fruit [4,5,6].

Selecting the proper rootstock is decisive in order to succeed in a commercial citrus fruit plantation [4]. Fruit quality is currently valued, in addition to visual attributes (e.g., size, color), including chemical properties such as contents of vitamins, minerals, carotenoids, phenols, and volatile compounds [7]. The organic compounds are associated with the fruit aroma and are present in peel, flowers, leaves, and juice [8]. In citrus species, the main quality characteristic is the aroma [9]. The quality of the lemon is highly influenced by the rootstock [10]. Several factors may modify the volatile profile of the lemon, including factors such as rootstock and variety [11,12,13]. Among these factors we can include environment, soil fertility, the content of beneficial microorganisms, the state of immaturity (green color), and unpeeled vs. peeled fruit juice [14,15,16,17]. Additionally, the volatile fraction may be altered by analytical method, sampling, and equipment used [18,19]. 

Throughout the world, citrus flavors are some of the most important flavors in the global market [20]. In this sense, fresh lemon peel can be used to obtain volatile compounds which give the characteristic citrus aroma and flavor [21,22]. For this, many citrus cultivars have been analyzed to identify their volatile profile [20]. Several authors have studied the volatile profile of the oil from the lemon peel [22,23,24], but information on how the rootstock influences the odorous compounds is very limited. 

Thus, the purpose of this study was to identify and quantify the volatile profile of five varieties of lemon grafted on five rootstocks and analyze the influence that the rootstock–graft interaction can have on the volatile profile of lemons.

## 2. Materials and Methods 

### 2.1. Plant Materials

Fruits were collected from 10-year-old, healthy trees, cultivated under the same pedoclimatic and cultural conditions. The climate was characterized by mild winters and slightly hot summers, temperatures ranging between 26 and 17 °C, and light rains concentrated in spring and autumn. Soil characteristics were as follows: sandy loam texture, 40% calcium carbonate, 8% active calcium, and pH = 8. The field was located at the IVIA (*Instituto Valenciano de Investigaciones Agrarias*) Experimental Station in Elche (latitude 38°14′56″ N, longitude 0°41′35.95″ E, altitude 149 m above sea level). 

The selected varieties of *Citrus limon* used in this study were “Betera”, “Verna”, “Fino 49”, “Fino 95”, and “Eureka”; grafted on to the rootstocks Forner-Alcaide N°5 (“FA5”), Forner-Alcaide N°13 (“FA13”), Forner-Alcaide N°517 (“FA517”), *C. macrophylla* West, and *C. aurantium* L. The progenitors of hybrids Forner-Alcaide N°5 (“FA5”) and Forner-Alcaide N°13 (“FA13”) were “Cleopatra” mandarin × *Poncirus trifoliata* (L.) Raf., both characterized as being resistant to salinity and tolerant to waterlogging. Forner-Alcaide N°517 (“King” (mandarin) × *P. trifoliata*) is distinguished by its tolerance to limestone and its dwarfing character. All of them reside in the European Union (BOE/04/12/2007) and were obtained by targeted hybridizations by Forner in IVIA (Valencia) [25].

Twenty-five plots (a combination of variety and rootstock) were used for this study, with a completely randomized factorial design. Each plot was composed of six trees spaced at 6 m × 2.5 m. 

Twenty lemons from each tree in each plot were collected. Next, the lemons were manually peeled with a peeler (no albedo was collected). Subsequently, the lemon peels were crushed with a grinder (Delhi model 180 W; Moulinex, Alençon, France) for 3 min and kept at −20 °C until analysis. 

### 2.2. Determination of Volatile Compounds

For the determination of the volatile compounds, the essential oil was extracted using the protocol described by El-Zaeddi et al. [26] with slight modifications. Hydrodistillation (HD) using a Deryng system was used for isolating the essential oil in the lemons. Sixty grams of crushed lemon skin was placed in a 500 mL round-bottom flask with 200 mL of distilled water and 200 µL of isoamyl acetate, which was used as an internal standard. Once the mixture was boiling for 5 min, 2 mL of the essential oil was collected in a vial of 2.5 mL and maintained in refrigerated storage (4 °C) until the gas chromatography-mass spectrometry (GC-MS) analyses were conducted. All the samples were extracted in triplicate. 

Volatile compounds were analyzed and identified using a Shimadzu GC-17A gas chromatograph coupled to a Shimadzu QP-5050A mass-spectrometry detector (Shimadzu Corporation, Kyoto, Japan). The GC-MS system was equipped with a Supelco (Supelco, Inc., Bellefonte, PA, USA) SLB-5 MS column (fused silica) 30 m × 0.25 mm, with a film thickness of 0.25 μm. The carrier gas used for this analysis was helium kept at a column flow rate of 0.6 mL min^−1^ and a total flow of 181.2 mL min^−1^ in a split ratio of 1:300. The program started with an increase of 3 °C min^−1^ from 80 to 170 °C. Afterwards, the temperature was increased at 25 °C·min^−1^ to 300 °C, maintaining this final temperature for 1 min. The temperature of the detector was 300 °C, and it was 230 °C for the injector. 

Three methods were used to identify volatile compounds: (1) retention rates and their comparison with those in the literature; (2) retention times of pure chemical compounds; (3) mass spectra of authentic chemical compounds and the spectral library of the National Institute of Standards and Technology (NIST) database. In this study, only fully identified compounds have been described. The analysis of the volatile composition was run in triplicate for each extraction and the results were expressed as the concentration of each of the volatile compounds as well as the concentration of the main chemical families of compounds.

### 2.3. Statistical Analysis

Two-way analysis of variance (ANOVA) and Tukey’s multiple range test were performed to compare experimental data and to determine significant differences among varieties and rootstock (*p* < 0.05). Principal component analysis (PCA) using Pearson correlation was also run. The software XLSTAT (Addinsoft 2016.02.270444 version, Paris, France) was used.

## 3. Results and Discussion

### 3.1. Identification of Volatile Compounds in Lemon Peels

Twenty-six volatile compounds in the lemon peel oils were identified by GC-MS (Table 1). These compounds can be grouped into four main chemical families: (i) monoterpenes (20 compounds); (ii) sesquiterpenes (3 compounds); (iii) aldehydes (2 compounds), and (iv) esters (1 compound). Moreover, Table 1 shows the main sensory descriptors of each of the volatiles identified in the lemon peel oils. 

### 3.2. Effects of Rootstock/Scion Combination in the Profile Volatile Compounds

Table 2 shows the concentration of the 26 compounds, expressed in mg·kg^−1^, identified and quantified in lemon peel oils. The order from the highest to lowest concentration was: limonene, β-pinene, γ-terpinene, sabinene, α-pinene, geranial, neral, α-thujene, β-bisabolene, terpinolene, *trans*-α-bergamotene, α-terpineol, α-terpinene, neryl acetate, linalool, *p*-cymene, citronellal, *trans*-caryophyllene, terpineol-4, nerol, camphene, nonanal, geraniol, octylaldehyde, α-phellandrene, and *cis*-sabinene-hydrate. These results agreed with those previously obtained by Gonzalez-Mas et al. [30], Liu et al. [31], Cano-Lamadrid et al. [32], and Tekgül and Baysal [23].

The volatile profile of the five varieties of lemon studied was dominated by only five monoterpene hydrocarbon compounds (in decreasing order): limonene, β-pinene, γ-terpinene, sabinene, and α-pinene (Table 2). The most abundant volatile compound found in all varieties was limonene, and this volatile compound ranged from 19.76 g·kg^−1^ (“Verna”) to 22.71 g·kg^−1^ (“Eureka”). Limonene was followed by β-pinene, the content of which ranged from 3.75 g·kg^−1^ (“Fino 95”) to 5.01 g·kg^−1^ (“Verna”), γ-terpinene from 3.22 g·kg^−1^ (“Fino 49”) to 3.84 g·kg^−1^ (“Verna”), sabinene from 0.61 g·kg^−1^ (“Fino 95”) to 0.85 g·kg^−1^ (“Verna”), and α-pinene from 0.64 g·kg^−1^ (“Fino 95”) to 0.79 g·kg^−1^ (“Verna”). Among the varieties, the highest concentration of total volatile compounds was found (in decreasing order) in “Eureka”, followed by “Bétera” > “Fino 95” > “Verna” > “Fino 49”. The essential oil composition of the current five varieties of lemon was similar to that reported by Di Vaio et al. [33], who analyzed the peel of 18 lemon cultivars, and by Lota et al. [34] who analyzed the peel and leaf essential oils of 15 species of mandarins. Another 15 monoterpene hydrocarbons which had not been previously identified in lemon peel were also identified and quantified, but at lower contents (<0.2 g·kg^−1^). Di Vaio et al. [33] only identified 5 monoterpene in 18 lemon cultivars studied compared with the 20 monoterpenes identified in the present study. These differences may be due to the extraction methods, among other factors. Lu et al. [19] showed that differences in the presence or absence of volatile compounds depend on the oil distillation process; there is a greater presence of oxygenated compounds when hydrodistillated and a higher concentration of terpene compounds when pressed cold.

The results showed that rootstock strongly affected the total volatile contents (Table 2). The rootstocks of the Forner-Alcaide series (“FA517”, “FA13”, and “FA 5”) showed the highest values of limonene and γ-terpinene (>22 g·kg^−1^ and >3.8 g·kg^−1^, respectively), while the lowest values were in *C. macrophylla* and *C. aurantium*. In general, the series Forner-Alcaide rootstocks induced a greater content of all the volatile compounds identified compared to the traditional *C. aurantium* and *C. macrophylla* rootstocks. The reason for these differences among the rootstock of the Forner-Alcaide series and the *C. aurantium* and *C. macrophylla* rootstock might be to do with the specific rootstock/scion combinations which affect citrus fruit aroma volatiles levels, and these qualities may be governed by the level of rootstock/scion compatibility, which obviously affects the translocation of water, nutrients, plant growth regulators, and photosynthetic assimilates through the graft union.

The sesquiterpenes were the second most abundant chemical group in the lemon peel (Table 2). Only three compounds were identified (in decreasing order): β-bisabolene, *trans*-α-bergamotene, and *trans*-caryophyllene. Furthermore, the rootstocks of the Forner-Alcaide series showed the highest content for these three sesquiterpenes, while the *C. macrophylla* and *C. aurantium* had the lowest.

Two aldehyde compounds were identified: nonanal and octanal. The aldehyde concentrations were in the range of 14.7 to 28.9 mg·kg^−1^ in the varieties grafted on “FA 517” and *C. macrophylla* respectively for nonanal, and ranged between 10.2 mg·kg^−1^ to 19 mg·kg^−1^ in the varieties grafted on *C. aurantium* and “FA 5” respectively for octanal (Table 2). 

Finally, regarding the esters, only one compound was identified: neryl acetate. No significant differences were observed in either the variety or the rootstock (Table 2).

In this study, we examined the effects of five rootstocks, three new in the Forner-Alcaide series, and two commercially important rootstocks (i.e., *C. aurantium* and *C. macrophylla*) on volatile compounds in the lemon peel oils of five varieties. The results indicate that the effect of rootstock on the volatile compounds is a rather complex phenomenon that greatly depends on specific interactions between the rootstock and each particular scion variety. Our results agreed with those reported by Benjamin et al. [4] in varieties of mandarins, Seker et al. [35] in the fruits of peach, and Wang et al. [12] in grapevines and in pistachios [36]—they all noted that rootstocks influenced the concentration and availability of volatiles. This could be explained by the fact that grafted plants generally increase the uptake of water and minerals due to the roots of rootstock or the compatibility of graft and canopy [37].

### 3.3. Principal Component Analysis

**Figure 1:**
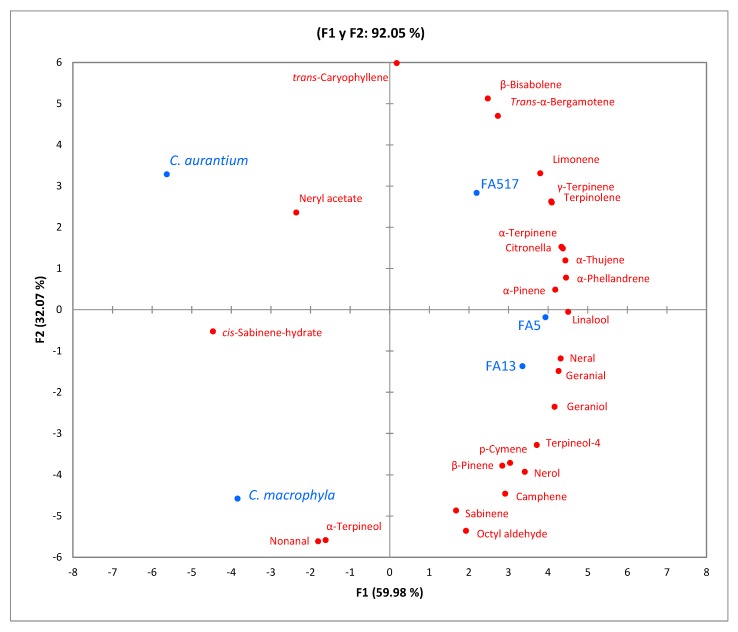
Principal component analysis (PCA) plot showing the relationships among volatile compounds and the factor “rootstock” (*n* = 9).

**Figure 2:**
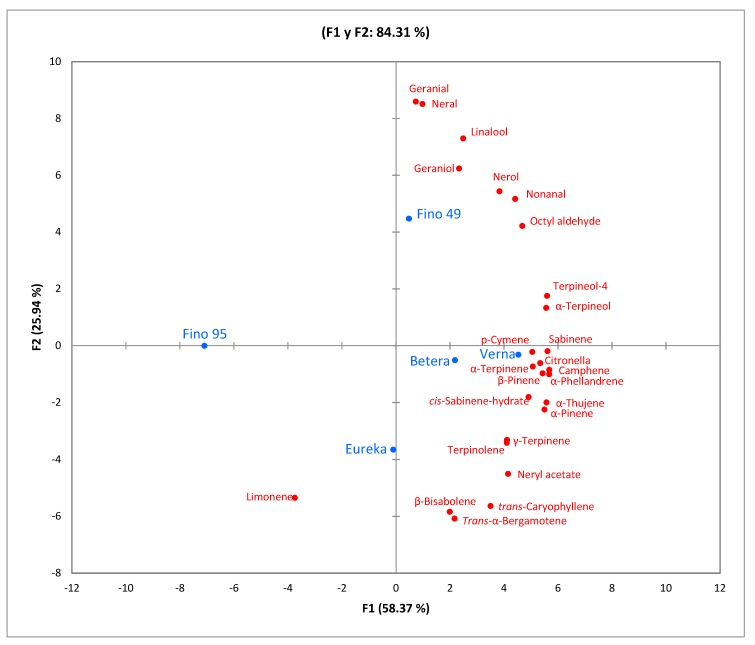
Principal component analysis (PCA) plot showing the relationship among volatile compounds and the factor “variety” (*n* = 9).

To better understand the relationships among the volatile compounds found (26 volatile compounds) in the different samples (varieties and rootstocks), principal component analyses (PCAs) were applied to the experimental results (Figure 1 and Figure 2). The PCA of the rootstocks (Figure 1) explained 92.05% of the variables in two axes, F1 (59.98%) and F2 (32.07%). Thanks to this statistical technique, it was very easy to observe that the *C. macrophylla* and *C. aurantium* rootstocks were isolated from the rest of the rootstocks, and were therefore characterized by volatile compounds such as nonanal and α-terpineol for *C. macrophylla* and neryl acetate in the case of *C. aurantium*. The rootstocks “FA517”, “FA5”, and “FA13” were linked to a higher number of volatile compounds, perhaps because genetically these rootstocks have a common parent and are characteristically smaller trees [25]. 

On the other hand, the PCA of the varieties (Figure 2) explained 84.31% of the variables in the F1 (58.37%) and F2 (25.94%) axes. This indicated that varieties such as “Betera”, “Verna”, and even “Eureka” had very similar aromatic profiles, while varieties such as “Fino 95” and “Fino 49” were isolated.

## 4. Conclusions

In this study, five rootstocks (three Forner-Alcaide rootstocks and two traditional *C. macrophylla* and *C. aurantium* rootstocks) were evaluated to study the effect on volatile composition of five commercial lemon varieties: “Bétera”, “Verna”, “Eureka”, “Fino 49”, and “Fino 95”. A total of 26 aromatic compounds were identified and quantified by GC-MS in lemon peel oils. Of all the aroma compounds identified in lemon peel oils, five monoterpene hydrocarbons (limonene, β-pinene, γ-terpinene, sabinene, and α-pinene) were present at the highest levels, followed by sesquiterpenes, aldehydes, and esters. The present experimental results demonstrate that Forner-Alcaide rootstocks (“FA5” > “FA517” > “FA13”) were the best rootstocks, leading to high content of volatile compounds, followed by *C. aurantium* and *C. macrophylla*. The order of total volatile contents was (in decreasing order): “Eureka” > “Bétera” > “Fino 95” > “Verna” > “Fino 49”. These results confirm that a strong relationship exists between the rootstock/scion combinations and the concentration of volatile compounds in the lemon peel oil. Aroma volatiles should be considered key parameters for the determination of rootstock-induced effects.

## Figures and Tables

**Table 1:** Retention indexes of the volatile compounds by GC-MS in lemon peel oils.

Compound		Chemical Family	Odor Properties	RT ^†^ (min)	KI (Exp.) ^‡^	KI (Lit.) *
1	α-Thujene	Monoterpene	Wood, green, herb ^⋆^	5.09	930	933
2	α-Pinene	Monoterpene	Pine, turpentine ^⋆^	5.28	939	944
3	Camphene	Monoterpene	Camphor ^⋆^	5.64	969	964
4	Sabinene	Monoterpene	Pepper, turpentine, wood ^⋆^	6.03	983	977
5	β-Pinene	Monoterpene	Pine, resin, turpentine ^⋆^	6.21	990	990
6	Octanal	Aldehyde	Strong and fruity smell ^๏^	6.62	1004	1001
7	α-Phellandrene	Monoterpene	Turpentine, mint, spice ^⋆^	6.78	1010	1003
8	α-Terpinene	Monoterpene	Lemon ^⋆^	7.04	1019	1018
9	p-Cymene	Monoterpene	Woody and spicy ^✠^	7.25	1027	1026
10	Limonene	Monoterpene	Lemon, orange ^⋆^	7.44	1034	1031
11	γ-Terpinene	Monoterpene	Gasoline, turpentine ^⋆^	8.15	1059	1062
12	*cis*-Sabinene-hydrate	Monoterpene	Herbal ^⋆^	8.64	1076	1074
13	Terpinolene	Monoterpene	Herbal ^⋆^	8.99	1089	1089
14	Linalool	Monoterpene	Flower, lavender ^⋆^	9.39	1103	1098
15	Nonanal	Aldehyde	Rancid ^✠^	9.51	1106	1102
16	Citronellal	Monoterpene	Fat ^⋆^	11.17	1152	1165
17	Terpineol-4	Monoterpene	Peppery, woody, sweet, musty ^⋆^	12.46	1189	1184
18	α-Terpineol	Monoterpene	Oil, anise, mint ^⋆^	13.02	1204	1197
19	Nerol	Monoterpene	Sweet ^⋆^	14.11	1231	1228
20	Neral	Monoterpene	Lemon ^⋆^	14.63	1244	1239
21	Geraniol	Monoterpene	Rose geranium ^⋆^	15.16	1257	1255
22	Geranial	Monoterpene	Lemon, mint ^⋆^	15.82	1273	1277
23	Neryl acetate	Ester	Fruit ^⋆^	19.43	1360	1366
24	*trans*-Caryophyllene	Sesquiterpene	Wood and spicy ^±^	22.18	1425	1420
25	*trans*-α-Bergamotene	Sesquiterpene	Wood ^⋆^	22.59	1435	1437
26	β-Bisabolene	Sesquiterpene	Balsamic ^⋆^	25.68	1509	1509

^†^ RT = retention time, ^‡^ KI (Exp.) = experimental Kovats indexes, * KI (Lit.) = literature Kovats indexes; ^⋆^ Tekgül and Baysal [23]; ^๏^ Lewis and Wiley [27]; ^✠^ Bravo et al. [28]; ^±^ Pino et al. [29].

**Table 2:** Concentrations (mg·kg^−1^) of volatile compounds in lemon peel oils.

	ANOVA ^†^			Variety					Rootstock				
Compound	V	R	V*R	Verna	Betera	Eureka	Fino 49	Fino 95	FA 5	FA 13	FA 517	*C. macrophylla*	*C. aurantium*
α-Thujene	***	***	***	195.1 a ^‡^	188.0 ab	170.0 bc	156.2 c	120.8 d	187.9 a	179.5 a	180.0 a	140.4 b	142.4 b
α-Pinene	***	***	***	797.3 a	765.8 ab	735.5 ab	704.8 b	648.5 b	798.1 a	743.8 ab	743.2 ab	685.5 b	681.2 b
Camphene	***	***	***	24.8 a	20.9 b	20.9 b	20.1 b	13.1 c	22.8 a	21.2 ab	18.9 b	21.4 ab	15.4 c
Sabinene	***	***	***	853.0 a	758.8 b	721.9 bc	730.3 bc	611.8 c	802.6 a	734.8 abc	691.9 bc	785.9 ab	660.5 c
β-Pinene	***	***	***	5011 a	4390 b	4417 b	4327 b	3757 b	4870 a	4451 abc	4193 bc	4501 ab	3888 c
Octanal	***	***	***	20.9 a	18.8 a	10.2 b	18.5 a	9.9 b	16.2 a	17.6 a	15.1 ab	19.0 a	10.2 b
α-Phellandrene	***	***	***	15.5 a	15.2 ab	14.6 ab	14.3 ab	11.7 b	16.0 a	15.4 a	15.9 a	12.3 b	11.7 b
α-Terpinene	***	***	***	101.0 a	98.1 ab	86.6 ab	87.1 ab	81.6 b	101.9 a	101.8 a	99.2 a	73.6 b	77.9 b
*p*-Cymene	NS	NS	***	88.1	80.0	88.5	86.6	55.8	85.6	92.0	72.9	79.6	68.9
Limonene	***	***	***	19,760 c	21,140 b	22,716 a	20,398 bc	22,107 ab	22,248 a	22,189 a	22,726 a	18,604 c	20,354 b
γ-Terpinene	***	***	***	3849 a	3786 a	3439 ab	3226 b	3299 ab	3967 a	3807 a	3882 a	2770 b	3172 b
*cis*-Sabinene-hydrate	***	NS	***	15.3 a	9.0 b	7.7 b	6.0 b	3.8 b	6.2	6.2	7.4	11.0	11.0
Terpinolene	***	***	***	167.8 a	166.2 a	151.5 ab	142.3 b	145.2 ab	175.5 a	167.6 a	171.2 a	119.9 b	138.8 b
Linalool	NS	NS	***	80.2	80.2	76.3	89.8	74.7	87.0	85.1	85.0	74.2	69.9
Nonanal	NS	**	**	21.1	21.4	17.8	23.9	14.8	19.0 ab	19.1 ab	14.7 b	28.9 a	17.4 b
Citronella	NS	***	***	74.1	76.7	68.3	68.5	60.0	85.3 a	77.2 a	85.4 a	49.7 b	50.0 b
Terpineol-4	*	NS	***	55.0 a	52.7 a	45.0 a	52.3 a	28.3 b	50.7	57.1	46.7	46.7	32.1
α-Terpineol	**	NS	***	141.5 a	141.7 a	116.8 ab	135.0 a	60.6 b	113.1	118.4	110.7	143.2	110.3
Nerol	NS	NS	**	38.4	38.0	33.4	51.1	22.3	42.6	44.5	37.5	40.3	18.3
Neral	NS	*	**	265.3	258.0	220.5	329.1	251.5	289.0 ab	317.0 a	287.3 ab	237.4 ab	193.5 b
Geraniol	NS	*	**	15.6	11.4	13.5	24.2	9.2	21.0 a	21.2 ab	14.5 ab	12.4 ab	3.8 b
Geranial	NS	NS	**	265.5	263.7	210.0	341.4	256.2	294.0	325.2	291.7	241.8	184.2
Neryl acetate	NS	NS	***	107.8	92.3	122.2	86.1	59.9	99.5	82.7	89.4	92.5	104.1
*trans*-Caryophyllene	***	***	***	64.8 ab	61.4 ab	75.8 a	55.2 b	46.9 b	61.5 ab	54.0 b	71.2 a	49.3 b	68.0 ab
*trans*-α-Bergamotene	***	***	***	139.0 b	142.5 ab	168.2 a	133.1 b	124.0 b	150.2 ab	138.3 bc	162.0 a	117.5 c	138.8 abc
β-Bisabolene	NS	***	***	170.8	174.6	203.9	167.1	156.1	185.1 a	171.9 ab	197.0 a	142.4 b	176.1 ab
Total	NS	***	***	32,338	32,851	33,950	31,473	32,029	34,796 a	34,037 a	34,310 a	29,100 b	30,399 b

^†^ NS = non-significant F ratio (*p* < 0.05); *, ** and *** significant at *p* < 0.05, 0.01, and 0.001, respectively. ^‡^ Values followed by the same letter within the same row were not significantly different (*p* < 0.05) according to Tukey’s least significant difference test (*n* = 9).
